# Correction: Can situations awaken emotions? The compilation and evaluation of the Emotional Situation Sentence System (ESSS)

**DOI:** 10.1371/journal.pone.0305649

**Published:** 2024-06-12

**Authors:** Yuan Zhao, Ming Yin, Chuanlin Zhu, Chenghui Tan, Shengjie Hu, Dianzhi Liu

There are errors in the author affiliations. The correct affiliations are as follows:

Yuan Zhao^1^, Ming Yin^2^, Chuanlin Zhu^3^, Chenghui Tan^4^, Shengjie Hu^3^, Dianzhi Liu^3^

**1** Police Officer Academy, Shandong University of Political Science and Law, Jinan, China, **2** Jiangsu Police Institute, Nanjing, China, **3** School of Educational Science, Yangzhou University, Yangzhou, China **4** School of Education, Soochow University, Suzhou, China

There are errors in the Funding statement. The correct Funding statement is as follows: This research is supported by the Qinglan Project of Jiangsu Universities, the police lie detection method with micro-expression recognition (Applied Innovation Project of Ministry of Public Security, Project No.: 2018YYCXJSST029); the Science and Technology Innovation Team of Jiangsu Police Institute, and the Program to Cultivate Middle-aged and Young Science Leaders of Colleges and Universities of Jiangsu Province, China.
