# Supplementary material for: Can situations awaken emotions? The compilation and evaluation of the Emotional Situation Sentence System (ESSS)
Source: PLoS One. 2021 Jul 19;16(7):e0252671. doi: 10.1371/journal.pone.0252671 (PMC8289053; doi:10.1371/journal.pone.0252671)
Supplement: S1 Text — (DOCX) [file pone.0252671.s001.docx]

**S1 Text. The emotion-inducing situations questionnaire.**

A. Please describe in one sentence a situation that makes you fear, such as “There was an explosion in a crowded place.”

B. Please describe in one sentence a situation that makes you disgust, such as “Walking down the street, I stepped in dog poop.”

C. Please describe in one sentence a situation that makes you anger, such as “My roommate plays computer games all night so that I can’t sleep.”

D. Please describe in one sentence a situation that makes you sad, such as “My beloved grandmother died suddenly.”

E. Please describe in one sentence a situation that makes you anxious, such as “Suddenly losing your Internet connection while playing with a teammate.”

F. Please describe in one sentence a situation that makes you happy, such as “The girl I like agreed with my confession.”

G. Please describe in one sentence a situation that makes you calm “neutral” that is, quiet, peaceful and calm, such as “Seeing a globe on your desk.”
